# Effects of a structured ergonomic training on knowledge and utilization of ergonomics among bankers – A single-blind pragmatic randomized controlled trial

**DOI:** 10.1177/10519815251334114

**Published:** 2025-04-21

**Authors:** Echezona Nelson Dominic Ekechukwu, Chisom Favour Ede, Stephen Sunday Ede

**Affiliations:** 1 Department of Medical Rehabilitation, Faculty of Health Sciences and Technology, College of Medicine, University of Nigeria, Enugu, Nigeria; 2 Environmental and Occupational Health Unit, Institute of Public Health, College of Medicine, University of Nigeria, Enugu, Nigeria; 3 LANCET Physiotherapy, Wellness and Research Centre, Enugu, Nigeria; 4 School of Sports and Health Sciences, University of Central Lancashire, Preston, UK; 5 Research Centre of Applied Sports and Physical Activity, University of Central Lancashire, Preston, UK

**Keywords:** banking, ergonomics, health education, knowledge, occupational health, posture, training programs, workplace

## Abstract

**Background:**

Poor ergonomic knowledge and its utilization is a significant risk factor for work-related musculoskeletal disorders reported to be high among bankers. Empirical evidence of an effective structured ergonomic training intervention on the knowledge and utilization (KU) of ergonomics for this population is lacking.

**Objectives:**

This study sought to determine the effectiveness of a structured ergonomic training intervention on the KU of ergonomics principles among bankers in Nigeria.

**Methods:**

This is a single-blind pragmatic randomized controlled trial with a cluster-randomization of 45 Nigerian bankers into intervention (structured ergonomic training - SET, n = 27) and control (placebo ergonomic training - PET, n = 18) groups. Ergonomic Knowledge/Utilization before (KU1) and after (KU2) training was assessed using a questionnaire developed through a two-step expert validation process. Data were analyzed descriptively and inferentially using independent and paired t-tests at α=^0.05^.

**Results:**

At baseline, the participants’ variables of age (28.52 ± 2.89years vs 28.72 ± 3.34years), BMI (25.14 ± 3.75 kgm^−2^ vs 23.45 ± 3.51kgm^−2^), and KU1(2.96 ± 1.65 vs 2.56 ± 1.72) of both groups (SET vs PET) were comparable (p > 0.05). However, the KU2 (t = 6.70, p < 0.0001) and change in KU (t = 7.05, p < 0.0001) were significantly higher in SET (7.33 ± 1.33and 4.37 ± 1.69 respectively) than in PET (2.93 ± 2.34 and 0.47 ± 1.77 respectively).

**Conclusion:**

This study finding revealed a low level of knowledge and utilization of ergonomic principles among Nigerian bankers and showed that structured ergonomic training protocol is effective in improving the knowledge and utilization of ergonomic principles among Nigerian bankers. A nationwide ergonomic training and evaluation using this training manual is recommended.

## Introduction

Most job tasks in the banking industry now require the use of technology especially personal computers for data entry. Advancements in technology in the form of electronic data and appliances have affected these workers and their working environment.^1^ The International Labor Organization has reported some worrying issues for workers in the financial industry that are related to increased digitalization and changes in work organization.^2^ Associated issues to these changes have been reported including greater pressure on time, reshaped interpersonal relationships and work processes,^3^ excessive work demands,^4^ difficult relationships with customers, and rising cases of mental stress.^5^

Since electronic data are mainly displayed on visual display terminals, improper body posture and prolonged sitting in front of these terminals are common and can lead to many health hazards such as eye strain,^6^ muscle fatigue, and other musculoskeletal discomforts.^7^ It is also reported to impact workers’ mental health,^5^ especially due to psychosocial stressors from these work-related musculoskeletal disorders (WRMSDs).^8^

These challenges from working with computers such as musculoskeletal disorders, stress, and their attendant consequences affect workers’ productivity, as well as the economy.^6,9^ The identified challenges can be addressed with a sound knowledge and utilization (KU) of the principles of ergonomics.^1,10^ The field of ergonomics aims to understand interactions among humans and other elements of a working system, to inform designs of jobs within normal human abilities and functions thereby optimize human well-being and overall system performance.^11,12^ For instance, Zakerian et al.^13^ showed in their cross-sectional study among bankers in Iran that ergonomic factors such as temperature, furniture, noise, and spatial arrangement of the workstation influenced workers’ productivity. Thus, an adequate ergonomic design and approach to work can help boost productivity while maintaining worker's optimal physical and mental health.^14,15^

Proper ergonomic training is an important intervention for preventing and eliminating the occurrence and progression of workplace injuries and stressors.^16^ Several studies^16–18^ have assessed the impact of varying ergonomic training interventions on work-related computer use with findings that support the use of ergonomic intervention programs in reducing ergonomic risk factors among computer workers. However, the literature is lacking on the impact of an ergonomic intervention specifically targeted at bankers, especially in a developing country like Nigeria.

Bankers in Nigeria are exposed to a high work pace in a bid to meet th**e** teaming customers and the economic target of the sector.^19,20^ This is in addition to reported unhealthy work practices such as working to meet targets in a constrained posture, job stress,^21^ musculoskeletal disorders, and job dissatisfaction.^22^ Previous literature has pointed out that workers in the Nigerian banking sector have poor KU of ergonomic principles.^14,23^ There appears to be no study in this environment that has assessed the effects of an ergonomic intervention among this cohort. This study therefore was aimed at determining the impact of structured ergonomic training (SET) intervention on the KU of ergonomics principles among bankers in Nigeria.

## Methods

### Research design

This single-blind pragmatic randomized controlled trial used cluster-randomization to recruit 45 bankers in Enugu state, Nigeria, who participated in the study and were thus randomized into two groups based on their bank branches – the experimental group (SET, n = 27) and the control group (placebo ergonomic training (PET), n = 18). The minimal sample size of 44 was determined using G*Power software (version 3.1) for a one-tailed t-test comparing the means of two independent groups with an effect size of 0.99 at a degree of freedom (dfb) = 42, to achieve 95% (0.95) power with a large effect size of 0.99 at an alpha level of 0.05. This large effect size was utilized to reduce the sample size to the minimum possible given the difficulty in recruiting banker given their busy work schedules.^24^ Thus, 57 bankers were initially recruited for the study but only 45 completed the study. The target populations for this study were both male and female professional bankers who actively participated in carrying out banking activities or tasks using a visual display terminal, working not less than 8 h daily in a minimum of 5 days per week, have worked for at least one calendar year and were willing to participate in the study. Those who were pregnant and those with known physical challenges like musculoskeletal disorders were excluded.

Ethical approval was obtained for this study from the health research and ethics committee of the University of Nigeria Teaching Hospital, Ituku Ozalla (UNTH) (NHREC/05/01/200BB-FWA00002458-1RB00002323). The procedure, ethical obligations, and the purpose of the research were clearly explained to participants who gave their written informed consent before the study commenced. All study protocols were conducted according to the Declaration of Helsinki.

### Variables and outcomes

Demographic and work features of the participants including biodata, job category, job duration, and anthropometric measures were assessed using a data form. The participants’ age was assessed to the last birthday in years, while their weight (kg) and height (m) were measured using the body composition monitor (Omron, BF508) and improvised stadiometer respectively. The body mass index (BMI) was then estimated for each participant as the body weight divided by the square of the body height in units of Kg/m^2^. More so, data on the job category was elicited to describe the nature of tasks routinely carried across the participants. The job duration was assessed as the period each participant has been on the job, which was calculated in years.

Also, their workstation assessment score (WSA) was evaluated using the University of Wyoming Workstation Ergonomic Assessment form^25^; while their General Health score and Mental Work score, and Job Stress score were assessed using their domains in the NIOSH Generic Stress Questionnaire.^26^

### Ergonomic knowledge and utilization (KU) questionnaire

**Figure 1.**
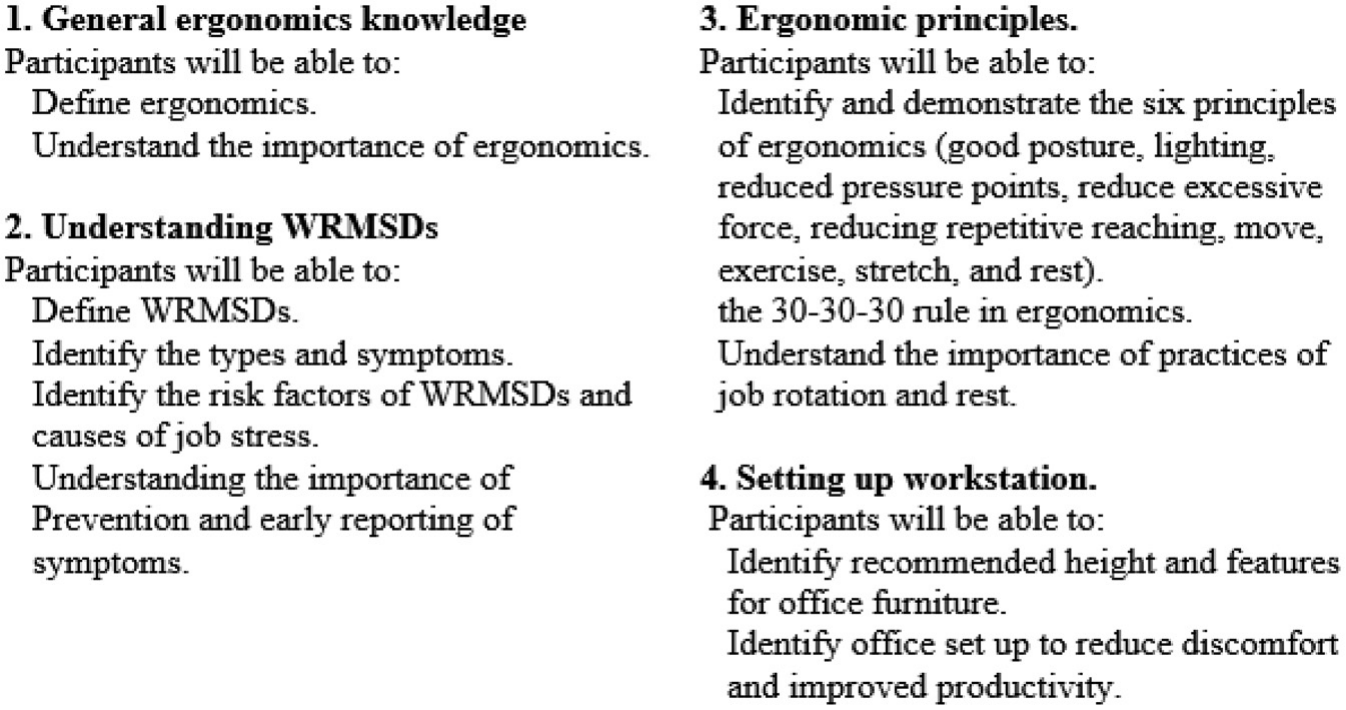
Ergonomic training curriculum outline for bank workers.

This tool was used before (KU1) and after (KU2) training for pre- and post-assessment. It contained 20 items, 10 each for KU. The items were statements of facts about ergonomic principles and practices like those in the training curriculum (Figure 1), for which the respondent was required to choose responses from a three-Likert scale of ‘agree, disagree, or don’t know’. Examples include: ‘‘My mouse should be at the same height as my keyboard’’ (knowledge item); ‘‘I include neck and wrist exercises in my daily work plan’’ (utilization item); ‘‘I type with my wrists bent slightly upwards’’ (utilization item); and ‘‘My computer desk should face a window for best lighting’’ (knowledge item). The posttest assessment utilized the same questions with some changes to reflect the respondent's intended practices. For example, the pretest item ‘‘I have evaluated my workstation for ergonomic hazards,’’ was changed on the posttest version to read, ‘‘I plan to evaluate my workstation for ergonomic hazards.’’

This Ergonomic Knowledge and Utilization Questionnaire was developed through a double-staged expert validation process having adapted the content from related previous studies among teleworkers.^27^ Seven subject matter experts (SME) who are knowledgeable in Human Factors/Ergonomics and instrumentation reviewed the test item for content validity by estimating its content validity index (CVI) using two steps. The first step involved the assessment of the essentiality of the items in the instrument. These SMEs were expected to score each item in the instrument a minimum of 0 and a maximum of 5 based on their perception of the usefulness/relevance of the item to the overall purpose of the instrument. The second step involved the extrusion of items with pooled scores below 3 and the calculation of content validity ratio (CVI) by summing up the overall score of the items and dividing by 5. Thus, the closer the index is to 1, the greater the content validity. The items that showed content validity of above 0.7 were included, which saw the reduction of the total items to 20 from an original 28.

### Ergonomic training manual

A self-developed and content-validated ergonomic handbook for training workers on ergonomic principles at various workplaces and specifically for the bankers’ population was drafted. The manual contained lessons on general ergonomics knowledge, understanding musculoskeletal disorders, best postures during computer usage, the principles of ergonomics, recommended height and features for office furniture, set-up of a computer workstation, and ergonomic stretching and exercises. It also covered the 30-30-30 rule in ergonomics, risk factors of WRMSDs, major causes of job stress in a workstation, and practices of job rotation and enlargement. These lessons were structured into a 45-min oral training with learning objectives and a detailed curriculum guide as shown in Figure 1, based on previous studies with teleworkers.^27^ The training was tailored for the bankers by incorporating their workstation setup for practical illustrations and hands-on demonstrations.

The content validity of this training manual was assessed by estimating its CVI through a two-step approach. The first step involved the assessment of the essentiality of the items in the instrument by a panel of 7 subject SME who are knowledgeable in Human Factors/Ergonomics and instrumentation. These SMEs were expected to score each item in the instrument a minimum of 0 and a maximum of 5 based on their perception of the usefulness/relevance of the item to the overall purpose of the instrument. The second step involved the extrusion of items with pooled scores below 3 and the calculation of content validity ratio (CVI) by summing up the overall score of the items and dividing by 5. Thus, the closer the index is to 1, the greater the content validity. The training manual had an excellent content validity (CVI = 0.85).

### Study procedure

A multi-stage sampling technique was utilized starting with a simple random sampling technique (fishbowl method) to select the participating banks among the populations of commercial banks in the Enugu metropolis. This was followed by purposive sampling of the bank workers for those who met the eligibility criteria and were willing to participate in the study. All participants from the same banks were allocated to the same group (cluster-randomized) to help reduce the contamination between the groups.

**Figure 2.**
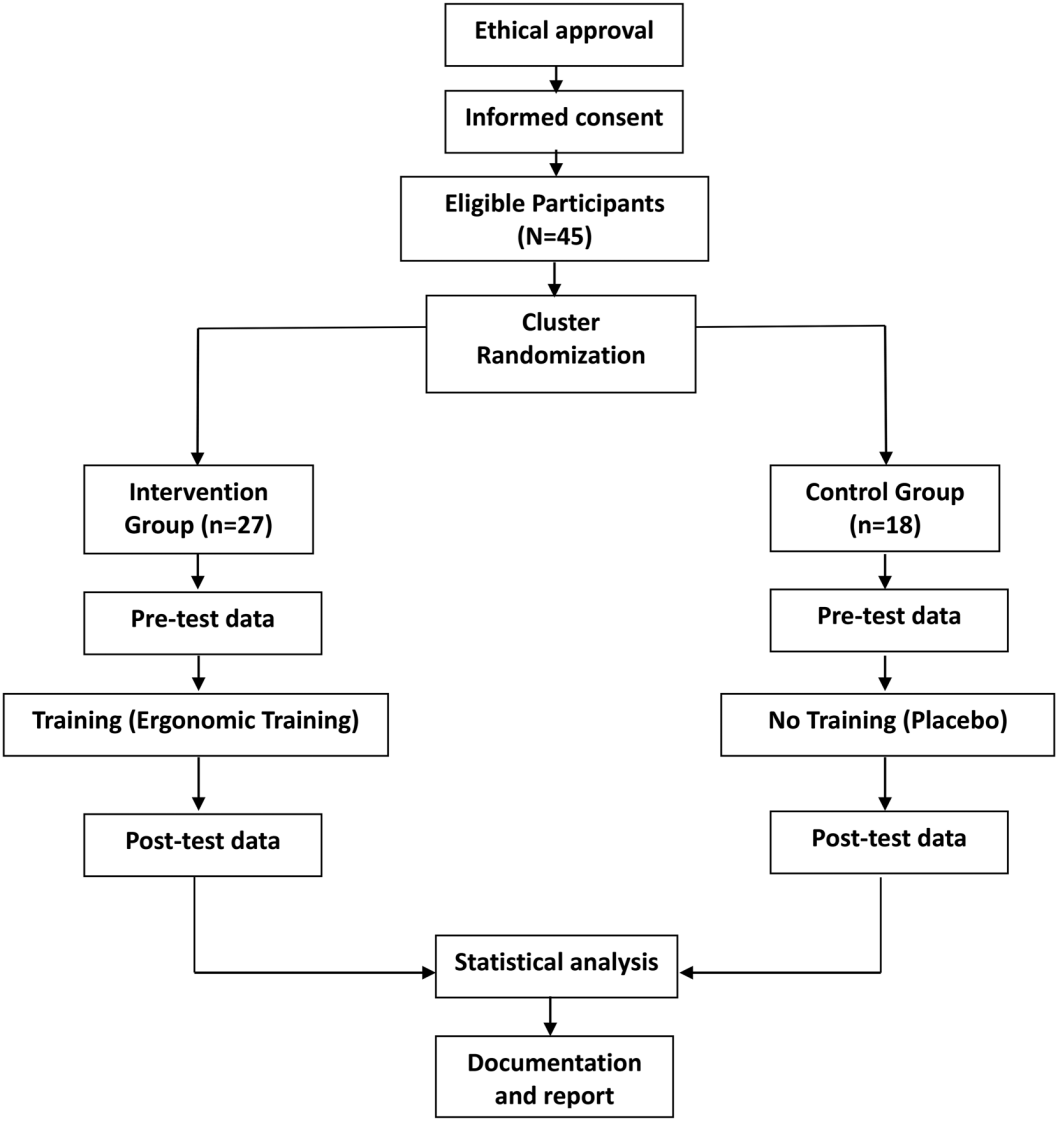
Schematic diagram of study design.

The study was carried out in the banking premises of the selected banks between March 2018 and January 2019. An introductory message was given explaining the purpose of the study to the participants and their informed consent was obtained. The pre-test assessments were done using the designated instruments. Thereafter, detailed ergonomic training was given to the bankers in the intervention group using the Ergonomic Training Manual, while a placebo training (introductory message, without ergonomic orientation) was given to the participants in the control group. The post-test assessment was done six weeks after the training. In addition, the control group received the same ergonomic training (as was given to the intervention group) after the study termination in line with ethical requirements as illustrated in Figure 2.

### Data analysis

Data obtained was analyzed using description statistics of mean and standard deviation, frequency, and percentages to summarize participants’ demographic variables and other description variables. Independent t-test was used to compare the mean of participant variables (that are normally distributed) between the intervention and control groups. Paired t-test was used to compare the pre-and post-test KU of ergonomic principles. Weighted regression analysis using inverse probability weighting was used to account for potential sampling bias due to unequal group sizes in our randomized study. The weighted estimates remained consistent with the unweighted results (β = 0.89, p = 0.37), indicating that the group imbalance did not introduce significant bias in our findings. All analysis was carried out with the aid of the Statistics Package for Social Sciences (SPSS) version 21. The level of significance was set as p = 0.05.

## Results

### Demographic and work features of the participants

The participants’ mean age was 28.60 ± 3.04 years, with a mean BMI of 24.47 ± 3.71Kg/m^2,^ and have been on their jobs for a mean duration of 26.02 ± 24.77 months. The majority of the participants were female (62.2%), single (57.8%), and had a Bachelor degree or its equivalent as their highest level of qualification (86.7%) as shown in Table 1.

**Table 1.** Frequency distribution of demographic and work features of the participants (N = 45).

Variable	Categories	Freq	% / Mean ± SD
Study Group	SET	27	60
	PET	18	40
Job Category	Teller	13	28.9
	Direct Sales Agent	12	26.7
	Retails	6	13.3
	Sales Service Agent	7	15.6
	Customer Service	3	6.7
	Loan Sales and Agent	4	8.9
Sex	Male	17	37.8
	Female	28	62.2
Marital Status	Married	19	42.2
	Single	26	57.8
Education	BSC	39	86.7
	MSC	6	13.3
Age (years)			28.60 ± 3.04
Weight (kg)			70.29 ± 9.45
Height (m)			1.70 ± 0.12
BMI (kg/m)2			24.47 ± 3.71

*SET = Structured Ergonomics Training Group, PET = Placebo Ergonomic Training (Control) Group, BMI = Body Mass Index, BSC = Bachelors degree, MSC **=********* *masters degree.*

### Baseline comparison of participants’ variables

At baseline (pre-test), the participants’ variables were comparable between the intervention (SET) and control (PET) groups. There was no significant difference in the participants’ mean age (t = −0.21, p = 0.829) and height (t = −045, p = 0.655), though those in the PET group were slightly older and taller than those in the SET group. Also, there was no significant difference in the weight (t = 1.27, p = 0.211), BMI (t = 1.52, p = 0.137) as well as the KU of ergonomics (t = 0.80, p = 0.430) between the participants in both groups, though the participants in SET group had higher values than those in PET group as shown in Table 2.

**Table 2.** Baseline comparison of participants variables (N = 45).

Variables	Mean ± SD			t	p
	Total (N = 45)	SET (n = 27)	PET (n = 18)		
Age (Years)	28.60 ± 3.04	28.52 ± 2.89	28.72 ± 3.34	−0.21	0.829
Weight (Kg)	70.29 ± 9.45	71.74 ± 10.16	68.11 ± 8.05	1.27	0.211
Height (m)	1.70 ± 0.12	1.70 ± 0.13	1.71 ± 0.12	−0.45	0.655
BMI (Kgm-^2^)	24.47 ± 3.71	25.14 ± 3.75	23.45 ± 3.51	1.52	0.137
KU1 (x/10)	2.80 ± 1.67	2.96 ± 1.65	2.47 ± 1.73	0.80	0.430

*SET = Structured Ergonomics Training Group, PET = Placebo Ergonomic Training (Control) Group, BMI = Body Mass Index, KU1 = Knowledge and Utilization of Ergonomics Pre-Training.*

### Mean distribution of participant variables

As shown in Table 3, the mean of general health score and mental work score for the combined group were 32.22 ± 9.49 and 8.71 ± 2.20 whereas for experimental and control were 32.04 ± 8.60, 9.15 ± 2.27 and 32.50 ± 10.95, 8.06 ± 1.98 respectively. The job stress score mean for the combined group was 8.48 ± 2.49, and 8.48 ± 2.49 for the experimental while 7.89 ± 2.42 for the control. Also, the sitting duration and computer time mean duration for the combined groups were 6.11 ± 2.50 and 6.13 ± 2.44, while for the experimental was 5.74 ± 2.64 and 5.67 ± 2.69 and for the control group was 6.67 ± 2.22 and 6.83 ± 1.86. The mean for the WSA was 15.67 ± 2.48 for the combined group, 13.00 ± 3.27 for the experimental and 11.61 ± 3.05 for the control group.

**Table 3.** Mean distribution of participants variables combined (N = 45), experimental (N = 27) and control (N = 18) group.

VARIABLES	COMBINED GROUPS (N = 45)				EXPERIMENTAL (n = 27)		CONTROL (n = 18)	
	Min	Max	ẋ	SD	ẋ	SD	ẋ	SD
Age (years)	24.00	37.00	28.60	3.04	28.52	2.89	28.72	3.34
Job Duration (years)	1.00	34.00	26.02	24.77	27.52	25.94	23.78	23.46
Number of previous jobs	0.00	19.00	6.22	28.74	1.59	4.58	13.17	44.96
Weight (Kg)	53.00	95.00	70.29	9.45	71.74	10.16	68.11	8.05
Hight (M)	1.53	1.97	1.70	0.12	1.70	0.13	1.71	0.12
BMI	16.30	36.17	24.47	3.71	25.14	3.75	23.45	3.51
General Health score	17.00	54.00	32.22	9.49	32.04	8.60	32.50	10.95
Job Stress score	3.00	13.00	8.24	2.45	8.48	2.49	7.89	2.42
Mental Work score	5.00	14.00	8.71	2.20	9.15	2.27	8.06	1.98
Sitting Duration (hours)	2.00	10.00	6.11	2.50	5.74	2.64	6.67	2.22
Standing Duration (hours)	0.00	10.00	3.27	2.58	3.85	3.05	2.39	1.29
Walking Duration (hours)	0.00	8.00	2.96	2.11	3.26	2.40	2.50	1.54
Computer Duration(hours)	2.00	10.00	6.13	2.44	5.67	2.69	6.83	1.86
Work Duration/day(hours)	5.00	20.00	12.44	3.22	13.00	3.27	11.61	3.05
Workdays per week	5.00	6.00	5.04	0.21	5.07	0.27	5.00	0.00
Number of Work Surface	3.00	8.00	5.18	1.23	5.52	1.31	4.67	0.91
Number of Keyboard	0.00	5.00	1.58	1.01	1.44	0.93	1.78	1.11
Number of Document holder	6.00	6.00	6.00	0.00	6.00	0.00	6.00	0.00
Number of Light	4.00	8.00	5.00	1.24	5.33	1.47	4.50	0.51
Number of Monitor	0.00	4.00	1.53	1.06	1.22	0.80	2.00	1.24
Number of Chair	1.00	10.00	8.49	2.35	8.11	2.83	9.06	1.21
WSA score	7.00	21.00	15.67	2.48	15.48	2.53	15.94	2.44
KU1 (x/10)	0.00	7.00	2.80	1.67	2.96	1.65	2.56	1.72
KU2 (x/10)	0.00	9.00	5.76	2.75	7.33	1.33	2.93	2.34
KU Difference (x/10)	−3.00	8.00	2.98	2.54	4.37	1.69	0.47	1.77

*BMI = Body Mass Index, WSA = Workstation Assessment, KU1 = Knowledge and Utilization of Ergonomics Pre-Training**,** KU2 = Knowledge and Utilization of Ergonomics Post-Training.*

The total mean pre-training knowledge of combined, experimental, and control were 2.80 ± 1.67, 2.96 ± 1.65, and 2.56 ± 1.72 respectively. Post training 5.76 ± 2.75, 7.33 ± 1.33 and 2.93 ± 2.34. knowledge difference 2.98 ± 2.54, 4.37 ± 1.69 and 0.47 ± 1.77 respectively.

### Within-group comparisons of knowledge and utilization of ergonomics

While there was a significant increase in the KU of ergonomics among the participants in the intervention (SET) group (t = −13.43, p < 0.0001), there was no significant increase in those of the control (PET) group (t = −0.22, p = 0.324) as shown in Table 4.

**Table 4.** Within group comparison of knowledge and utilization of ergonomics (N = 45).

Group	KU1(x/10)	KU2 (x/10)	t	p	95% CI	
					Lower	Upper
SET	2.96 ± 1.65	7.33 ± 1.33	−13.43	<0.0001*	−5.04	−3.70
PET	2.47 ± 1.73	2.93 ± 2.34	−0.22	0.324	−1.45	0.51

*SET = Structured Ergonomics Training Group, PET = Placebo Ergonomic Training (Control) Group, KU1 = Knowledge and Utilization of Ergonomics Pre-Training, KU2 = Knowledge and Utilization of Ergonomics Post-Training.*

### Between-group comparisons of knowledge and utilization of ergonomics

There was a significant difference in the post-training KU of ergonomics when compared between the intervention (SET) group and the control (PET) group (t = 6.70, p < 0.0001). Also, there was a significant difference in the change in KU of ergonomics when compared between the groups (t = 7.05, p < 0.0001) as shown in Table 5.

**Table 5.** Between group comparisons of knowledge and utilization of ergonomics.

Variables	Mean ± SD			t	p
	Total (N = 45)	SET (n = 27)	PET (n = 18)		
KU2 (x/10)	5.76 ± 2.75	7.33 ± 1.33	2.93 ± 2.34	6.70	<0.0001*
ΔKU (x/10)	2.98 ± 2.54	4.37 ± 1.69	0.47 ± 1.77	7.05	<0.0001*

*SET = Structured Ergonomics Training (Intervention) Group, PET = Placebo Ergonomic Training (Control) Group, KU2 = Knowledge and Utilization of Ergonomics Post Training, ΔKU = Change in Knowledge and Utilization of Ergonomics (KU2-KU1).*

## Discussion

This study assessed the effects of SET intervention on the KU of ergonomics principles among bankers in Nigeria. The finding supported the study hypothesis, as there was a low level of KU of ergonomic principles among these cohorts prior to the training. These became significantly improved after the SET as assessed in a six-week follow-up.

The finding of low KU of ergonomic principles (less than the median score) among Nigerian bankers is consistent with earlier studies.^14,21–23,28^ In more recent work in Nigeria, Asogwa and Ndubuisi-Okolo^14^ similarly reported that ergonomic knowledge was very poor among bankers. There are often issues with workplace layout, repetitive tasks, and insufficient rest breaks needing interventions to enhance conducive environments and well-structured tasks.^14^ The report on low KU of ergonomic principles is also found in other developing nations. For instance, Sirajudeen et al.^29^ in their study on the assessment of knowledge of ergonomics among Indian technology, professionals reported similarly low knowledge among their participants. Although this study did not specifically target bankers, the findings suggest a broader trend of insufficient ergonomic knowledge among professionals in India, which could extend to the banking sector. A study by Kawade et al.^30^ assessed the knowledge and awareness of WRMSDs and office ergonomics among bankers in the Maval region of India. The findings revealed that while a significant number of bankers were aware of WRMSDs and office ergonomics, there was a substantial gap in ergonomic practices during their work tenure. This discrepancy suggests that despite awareness, practical application of ergonomic principles is lacking, leading to potential health issues and decreased efficiency. These studies collectively underscore the critical need for structured ergonomic programs in these categories of employees.

The low KU of ergonomic principles noted in this study are despite the relatively young age of the participants and the increasing influence of the internet/social media in making information accessible. Previous studies have identified factors such as the lack of understanding and exposure to the benefits derived from the application of ergonomic principles.^7,10,23,31^ It may also be due to resource constraints and the high cost of providing ergonomic materials for workers,^32^ which may not be available to small or medium-scale enterprises in Nigeria. This could mean that deliberate action is needed to spread this vital information.

This study further created and tested SET to bridge the low knowledge and utilization of ergonomic principles in Nigeria. This was compared in a pre-and post-training experimental and placebo group trial, which showed positive significant changes in the participants’ KU of ergonomic principles. Given the baseline uniform distribution of the participants, the significant difference in post-training values suggests that SET is effective in improving the KU of ergonomics principles among bankers. While research that has attempted a randomized controlled trial with a structured ergonomic program among bankers is scarce, the finding on the usefulness of SET in helping workers implement basic ergonomics principles and reducing the risk of musculoskeletal disorders is not novel. Studies in other populations regarding ergonomic workplace and training intervention have been reported.^10,17,18,33,34^ For instance, an earlier interventional follow-up study in Iran by Motamedzadeh et al.^10^ implemented both educational and physical interventions among 277 bank employees. After nine months, significant reductions in Rapid Office Strain Assessment scores and WRMSD prevalence, particularly in the neck, shoulders, and lower back, were observed in the intervention groups. A more recent study by Ağar et al.^34^ also validated these findings in their study on the impact of ergonomics training on office workers’ exposure to WRMSDs. The results highlighted the importance of educational interventions in promoting better ergonomic practices among employees.

The present study demonstrated potential suitability and applicability in resource-limited settings such as Nigeria, as training interventions aimed at enhancing knowledge and practices are both cost-effective and feasible. The training was implemented orally and can be completed within an hour, suitable to fit into new employees’ orientation. Further research with extended follow-up periods is necessary to validate the long-term adherence to and sustainability of ergonomic principles in practice.

Hence, this study finding suggests and recommends that SET will be a helpful tool in improving the knowledge of ergonomics among bankers. Mahmud et al.^35^ in their study shares a similar view as they noted that office ergonomic training could be effective in increasing ergonomic knowledge. Consequently, Momodu et al.^36^ later advocated for regular ergonomic education and awareness of ergonomics to be consciously taken to the door of employers and employees by the Ergonomic Society of Nigeria. This recommendation is reemphasized in this study given the huge implication of poor ergonomic practice on the musculoskeletal health of the workers as well as the benefits of working in an efficient and effective ergonomic environment.

### Limitations

Some limitations are noted in the study that should be cautiously considered when interpreting the findings. First, the study relied solely on knowledge assessments and self-reported behaviors, without incorporating physical or performance-based outcome measures. As a result, actual improvements in musculoskeletal health or workplace efficiency could not be objectively evaluated. There is the possible effect of response bias from the participants during the post-training data collection that might result in discrepancies in their actual practice and those reported. Secondly, the follow-up period was relatively short (only six weeks), making it uncertain whether the observed improvements in KU are sustained over time. The study also had a high attrition (21%), which could introduce bias to the findings. Future research can improve on these study findings by using observatory research methods to capture the actual practice of the bankers in the data collection and increasing the number of participating banking institutions to capture design and differences in their settings.

## Conclusions

The study demonstrated the effectiveness of a structured ergonomic intervention designed to reduce exposure to WRMSDs and improve work efficiency and productivity. This pragmatic randomized controlled trial revealed a low level of KU of ergonomic principles among Nigerian bankers at baseline. At a six-week follow-up, the finding showed that the SET protocol is effective in improving the KU of ergonomic principles in this population. This training protocol could potentially inform ergonomic orientation programs for bankers. Seminars and conferences targeted at impacting employees and employers with accurate knowledge and practices of ergonomics principles are recommended.

## References

[1] Nesindande AR Saurombe MD Joseph RM . Exploring changes in banking workplaces because of digital technology implementation. SA J Hum Resour Manag 2024; 22: 1–2.

[2] International Labour Organization. Digitalization and the future of work in the financial services sector: Issues paper for the technical meeting on the impact of digitalization in the finance sector (Geneva), https://www.ilo.org/sites/default/files/2024-08/Digitalization%20and%20the%20future%20of%20work%20in%20the%20financial%20services%20sector.pdf (24–28 January 2022, accessed 15 February 2025).

[3] Carreri A Gosetti G Masiero N . Digitalization of relational space in the service triangle: the case study of retail banking. Front Sociol 2023; 8: 1141879. 37066067 10.3389/fsoc.2023.1141879 PMC10101322

[4] Umans T Kockum M Nilsson E , et al. Digitalisation in the banking industry and workers subjective well-being: contingency perspective. Int J Workplace Health Manag 2018; 11: 411–423.

[5] Kulkarni P Appasaba LV Nishchitha GC . The influence of COVID-19 on employee ergonomics and employee engagement of banking employees. Manag Matters 2022; 19: 13–29.

[6] Nepal A Koirala R . Impact of ergonomics practices on commercial banks’ employee performance in Nepal: evidence from structural equation modeling. Quest J Manag Soc Sci 2024; 6: 175–194.

[7] Demissie B Bayih ET Demmelash AA . A systematic review of work-related musculoskeletal disorders and risk factors among computer users. Heliyon 2024; 10: e25075. 10.1016/j.heliyon.2024.e25075 PMC10840111 38318034

[8] Gogoi M Kalita M . Assessment of occupational health hazards of the computer users. J Ergon 2022; 12: 308.

[9] Ibrahim BA Gaafar SE . Work-related musculoskeletal complaints: risk factors and impact on work productivity among university administrative employees. J Egypt Public Health Assoc 2024; 99: 10. 38744733 10.1186/s42506-024-00156-w PMC11093958

[10] Motamedzadeh M Jalali M Golmohammadi R , et al. Ergonomic risk factors and musculoskeletal disorders in bank staff: an interventional follow-up study in Iran. J Egypt Public Health Assoc 2021; 96: 34. 34894327 10.1186/s42506-021-00097-8 PMC8665913

[11] Institute for Occupational Safety and Health (National Institute for Occupational Safety and Health). Ergonomics and musculoskeletal disorders, https://www.cdc.gov/niosh/topics/ergonomics/default.html (2012, accessed 20 November 2022).

[12] International Ergonomic Association (IEA). What is ergonomics (HFE)?, https://iea.cc/what-is-ergonomics/Retrieved (2013, accessed 20 November 2022).

[13] Zakerian SA Garosi E Abdi Z , et al. Studying the influence of workplace design on productivity of bank clerks. J Health Saf Work 2016; 6: 35–42.

[14] Asogwa OS Ndubuisi-Okolo PU . Effect of ergonomic factors on employees’ performance in Nigeria's banking sector. Eur J Bus Manag 2020; 12: 86–98.

[15] Judijanto L Sihite M Hanafie A , et al. The effect of work environment on employee productivity in the banking industry in Indonesia: a case study on ergonomics, mental health, and operational efficiency. West Sci J Econ Entrep 2023; 1: 309–318.

[16] de Waal A Killian A Gagela A , et al. Therapeutic approaches for the prevention of upper limb repetitive strain injuries in work-related computer use: a scoping review. J Occup Rehabil 2024; 6: 1–34. 38844712 10.1007/s10926-024-10204-z PMC12089234

[17] Hoe VC Urquhart DM Kelsall HL , et al. Ergonomic interventions for preventing work-related musculoskeletal disorders of the upper limb and neck among office workers. Cochrane Database Syst Rev 2018; 10: 1465–1858. 10.1002/14651858.CD008570.pub3 PMC6517177 30350850

[18] Lee S De Barros FC De Castro CS , et al. Effect of an ergonomic intervention involving workstation adjustments on musculoskeletal pain in office workers—a randomized controlled clinical trial. Ind Health 2021; 59: 78–85. 33250456 10.2486/indhealth.2020-0188 PMC8010160

[19] Tella BA Akodu AK Fasuba OO . The prevalence of neck and upper extremity repetitive stress injury among bank workers in Lagos, Nigeria. Internet J Rheum 2011; 6: 1–6.

[20] Kayode AA Adewale JA Lawal NTA . An exploration of prevalence of repetitive stress injuries among computer operators in Nigeria. Int J Comput Appl 2015; 109: 1–8.

[21] Eberendu IF Ozims SJ Agu GC , et al. Workplace health risks associated diseases and health promotion in the Nigerian banking sector. Int J Adv Res Biol Sci 2018; 5: 197–208.

[22] Okwor TJ Ndu AC Arinze-Onyia SU , et al. Prevalence and predictors of stress among bankers in Enugu State South-East Nigeria. J Community Med Prim Health Care 2020; 32: 68–79.

[23] Tella BA Akinfeleye AM Oghumu SN , et al. Association of complaints of arm, neck, and shoulders with physical and psychosocial risks factors among computer users of Nigerian bank employees. J Int Soc Phys Rehabil Med 2021; 4: 82–89.

[24] Serdar CC Cihan M Yücel D , et al. Sample size, power and effect size revisited: simplified and practical approaches in pre-clinical, clinical and laboratory studies. Biochemia Medica 2021; 31: 27–53. 10.11613/BM.2021.010502 PMC7745163 33380887

[25] University of Wyoming. Workstation ergonomic assessment form [Internet]. Laramie, WY: University of Wyoming, https://www.uwyo.edu/safety/occupational/docs/ergonomic-workstation-assessment-form.pdf (2024, accessed 17 February 2025).

[26] Hurrell JJ Jr McLaney MA . Exposure to job stress: a new psychometric instrument. Scand J Work Environ Health 1988; 14: 27–28. 3393871

[27] Harrington SS Walker BL . The effects of ergonomics training on the knowledge, attitudes, and practices of teleworkers. J Saf Res 2004; 35: 13–22. 10.1016/j.jsr.2003.07.002 14992842

[28] Ezema CI Amaeze AA . Assessment of office furniture and knowledge of work ergonomics among bank workers in Enugu metropolis. J Coll Med 2011; 16: 13–16.

[29] Sirajudeen MS Pillai PS Vali GMY . Assessment of knowledge of ergonomics among information technology professionals in India. Int J Health Rehabil Sci 2013; 2: 192–197.

[30] Kawade P Tembhurne S Ghodey S . Knowledge and awareness of work-related musculoskeletal disorders and office ergonomics among bankers in Maval region. Indian J Physiother Occup Ther 2022; 16: 113–120.

[31] Abdollahpour N Helali F Rasoulzadeh Y , et al. Barriers and challenges to human factors/ergonomics knowledge transfer to small business enterprises in an industrially developing country. IISE Trans Occup Ergon Hum Factors 2023; 11: 14–31. doi:10.1080/24725838.2023.2179687 36866842

[32] Olabode SO Adesanya AR Bakare AA . Ergonomics awareness and employee performance: an exploratory study. Econ Environ Stud 2017; 17: 813–829.

[33] Ahmadi Charkhabi S Motamedzade M Dianat I , et al. Investigation of the multi-component ergonomics intervention effects on improving musculoskeletal outcomes and speech communication: a case study in open-plan offices. Work 2023; 76: 275–288. 36872832 10.3233/WOR-220427

[34] Ağar A Yeginoğlu G Kızıltan B . The effect of ergonomics training given to office workers on musculoskeletal disorders and working postures. Int J Occup Saf Ergon 2025: 1–8. 10.1080/10803548.2025.2457186 39957568

[35] Mahmud N Kenny TD Zein RM , et al. The effects of office ergonomic training on musculoskeletal complaints, sickness absence, and psychological well-being: a cluster randomized control trial. Asia Pac J Public Health 2011; 20: 1–17. 10.1177/1010539511419199 21878465

[36] Momodu B Edosomwan J Edosomwan TO . Evaluation of ergonomics deficiencies in Nigerian computer workstations. J Ergon 2014; 4: 008.

